# Bacteria–phage infection network structure and genomic defence system content predict efficacy of a phage therapy cocktail against *Pseudomonas aeruginosa* from chronic lung infections

**DOI:** 10.1098/rstb.2024.0080

**Published:** 2025-09-04

**Authors:** Maisie R. Czernuszka, Taoran Fu, Anastasia Kottara, Michael A. Brockhurst, Rosanna C. T. Wright

**Affiliations:** ^1^ Division of Evolution, Infection and Genomics, University of Manchester, Manchester M13 9PT, UK; ^2^ Manchester Institute of Biotechnology, University of Manchester, Manchester M1 7DN, UK; ^3^ Pathogen Genomics and Virology Laboratory, UKHSA, Manchester M13 9WL, UK

**Keywords:** phage therapy, *Pseudomonas aeruginosa*, chronic infection, bacteria–phage infection network, anti-phage defence system

## Abstract

*Pseudomonas aeruginosa* chronic lung infections pose serious challenges for phage therapy due to high between-patient strain diversity and rapid within-patient phenotypic and genetic diversification, necessitating simple predictors of efficacy to streamline phage cocktail design. We quantified bacteria–phage infection networks (BPINs) for six phages against 900 *P. aeruginosa* clones previously isolated from 10 bronchiectasis infections (*n* = 90 isolates per patient). BPIN structure varied extensively between patients. The efficacy of the six-phage cocktail against these diverse *P. aeruginosa* populations was influenced by several factors. Cocktail efficacy increased with decreasing number and strength of individual resistances, as well as with increasing co-resistance modularity and phage dose. These results highlight simple BPIN metrics that could help guide the design of effective phage therapeutics. Resistance against some but not all the phages increased with higher number defence systems per genome, resulting in lower efficacy of the six-phage cocktail, suggesting that *P. aeruginosa* strains with fewer defence systems are better candidates for phage therapy. Overall, our findings suggest that ‘off the peg’ phage therapeutics are unlikely to be broadly effective against *P. aeruginosa* chronic respiratory infections, but that the design of personalised phage cocktails could be guided using simple BPIN metrics, and that defence systems per genome provide a useful rule of thumb for identifying highly treatable infections.

This article is part of the discussion meeting issue ‘The ecology and evolution of bacterial immune systems’.

## 1. Introduction

Phage therapy, using viruses that specifically infect and kill bacteria, is a promising alternative for treating drug-resistant bacterial infections [1]. However, phage therapy remains experimental in most countries and is primarily used as a last resort for compassionate cases [2]. The process of identifying appropriate phages for compassionate use by screening patient bacterial isolates against phage banks offers a tailored yet laborious and time-consuming approach to treatment [3,4]. This often involves large-scale *in vitro* plaquing assays to select phages that demonstrate strong lytic activity against patient-specific isolates; however, we currently lack a consensus framework for methodology and interpretation of results [5,6]. In contrast, some clinical trials are now exploring ‘off-the-peg’ phage cocktails with broad host ranges that could be deployed more rapidly and at scale [7,8]. The relative benefits of these two approaches, personalized versus standardized, remain debated, particularly for infections with high strain diversity where fixed cocktails may be less effective [9,10]. Much current research, therefore, seeks to identify predictors of phage infectivity and treatment efficacy to streamline treatment design, for example combining genomic data with bacteria–phage infection networks to identify genetic determinants of compatible bacteria–phage interactions [11,12]. This approach has shown that genetic determinants of infection can be identified in both phage and bacterial genomes, and that potentially effective phage treatments against major epidemic clones can be identified [11,12].

Here, we target chronic respiratory infections caused by *Pseudomonas aeruginosa*, which pose additional challenges for phage treatment design. *Pseudomonas aeruginosa* is an opportunistic bacterial pathogen and common cause of damaging lung infections in cystic fibrosis (CF) and bronchiectasis. In both diseases, *P. aeruginosa* infection is associated with driving inflammation, recurrent exacerbations, progressive lung damage and a higher chance of hospitalization [13–15]. In addition, *P. aeruginosa* infections readily evolve resistance to a wide range of clinical antibiotics, highlighting the urgent need for novel therapeutics [16]. In recent years, phage therapy has been used to treat *P. aeruginosa* infections in a growing number of compassionate use cases and clinical trials worldwide, including in respiratory infections, although efficacy remains variable and context-dependent [7,17,18]. Genomic analyses of both CF and bronchiectasis infections show that while epidemic clones, such as the Liverpool Epidemic Strain [19,20], do exist most patients typically acquire a unique strain [21,22]. The resultant high between-patient strain diversity poses a challenge for phage therapy, implying it will be unlikely for strategies using ‘off-the-peg’ phage treatments that target common epidemic clones to be effective against these infections. A second challenge for phage therapy is that in both CF and bronchiectasis infections, *P. aeruginosa* populations typically undergo extensive genetic and phenotypic diversification as they adapt to the lung environment [23,24]. This diversification often includes mutations in traits linked to phage interactions, such as loss-of-function mutations in genes associated with bacterial motility structures or cell envelope, which may interfere with phage adsorption and susceptibility [22,25], potentially making phage treatment less effective. A third challenge for phage therapy is that *P. aeruginosa* genomes encode a high diversity of anti-phage defence systems, with both the number and complement of defence systems varying extensively between genomes [26]. The accumulation of more defence systems per genome has been shown to protect against a wider range of individual phages in *P. aeruginosa* [27], but how defence systems affect the efficacy of phage cocktails remains poorly understood.

In this study, we quantified bacteria–phage infection networks (BPINs) and co-resistance networks for six diverse lytic phages that target two distinct cell-surface receptors (type IV pilus or lipopolysaccharide) against 900 *P*. *aeruginosa* clinical isolates previously isolated from 10 bronchiectasis patients (*n* = 90 isolates per patient) [22]. The structure of these networks varied substantially between patients, demonstrating both between and within patient diversity in phage susceptibility. Next, we reconstituted the within population diversity of infections by pooling all 90 isolates per patient and subsequently experimentally treated these diverse populations *in vitro* with a cocktail of all six phages across a range of dosages (on a log_10_ scale from multiplicity of infection 0.01–100). Treatment efficacy varied significantly between patients and was strongly influenced by the structure of the BPIN and co-resistance network within each patient. Finally, we analysed how the anti-phage defence system complements of a subset of *P. aeruginosa* strains per patient related to phage resistances and phage cocktail efficacies. Phage resistance increased with defence system number for four of the six phages, whereas the efficacy of the phage cocktail declined with increasing defence system number. Taken together, these findings highlight the importance of accounting for between and within patient diversity in phage resistance network structures when designing phage therapies against *P. aeruginosa* chronic infections and identify simple network metrics that could guide cocktail design. Moreover, we show that *P. aeruginosa* strains with smaller anti-phage defence arsenals are likely to be good candidates for phage therapy.

## 2. Methods

### (a) Bacterial strains and culturing techniques

*Pseudomonas aeruginosa* populations were previously cultured from sputum samples taken from 10 bronchiectasis patients and 90 colonies per patient were randomly chosen, of which 16 clones per patient were whole genome sequenced and analysed for defence system content [22]. Bacterial sequence types were distinct between patients, but all sequenced isolates from each patient contained the same sequence type [22]. Glycerol stocks of each set of 90 colonies were prepared in 96-well plates along with the control strains PAO1, LESB58 and ATCC27853, and stored at −80°C [22]. Ten of these *P. aeruginosa* populations were randomly chosen for investigation in this study. We did not have access to any clinical data for these patients for use in this study. These were grown in 200 µl of King’s Media B (KB) in 96-well plates for 48 h at 37°C in a static humidity incubator (75% humidity). Overnight cultures of PAO1 were prepared from glycerol stocks and inoculated in 6 ml KB in glass microcosms at 37°C in an orbital shaking incubator (180 r.p.m). Bacterial densities were quantified by plating serial dilutions on 1.2% solid KB agar plates and counting colony-forming units (CFU/ml^−1^) following incubation overnight at 37°C.

### (b) Phage strains and culturing techniques

Six lytic phages were selected based on their ability to infect *P. aeruginosa* PAO1 (see table 1). PELP20 was originally isolated from the Georgian Pyophage cocktail (Eliava Biopreparations Ltd, Georgia). The remaining five phages were isolated from various environmental/sewage water systems [28,29]. Fresh phage stocks were made by introducing frozen phage stocks into microcosms with 6 ml KB and 60 µl PAO1 overnight culture and were incubated at 37°C in a shaking orbital incubator (180 r.p.m) [30]. Phage stocks were purified by filtration using a 0.22 μm filter and kept at 4°C. Phage densities were quantified bi-weekly by spotting serial dilutions on a confluent lawn of PAO1 growing in a 0.6% soft agar overlay on a KB agar plate and calculating plaque-forming units (PFU/ml^−1^) following overnight incubation at 37°C.

**Table 1.** Summary of phage strains.

phage strain	source	date first isolated	known adsorption target	genome size (kb)	genus	accession number	reference
PELP20	phage cocktail (pyophage), Tbilisi, Georgia	information unavailable	lipopolysaccharide binding	66.19	*Pbunavirus*	unpublished	—
PA1P3	sewage water, Jyväskylä, Finland	2015	lipopolysaccharide binding	66.58	*Pbunavirus*	unpublished	[28]
PA8P1	sewage water, Jyväskylä, Finland	2015	lipopolysaccharide binding	65.82	*Pbunavirus*	NC_048806.1	[28]
PA5P2	sewage water, Jyväskylä, Finland	2015	type IV pilus binding	93.9	unknown	unpublished	[28]
PNM	Mtkvari River, Tbilisi, Georgia	1999	type IV pilus binding	42.72	*PhiKMVvirus*	OP292288.1	[29]
⏀KZ (phiKZ)	sewage water, Kazakhstan	1975	type IV pilus binding	280.33	*PhiKZvirus*	NC_004629.1	[29]

### (c) Clinical isolate phage resistance screening

To determine phage resistance of *P. aeruginosa* clones, phage were embedded in a soft agar (0.6% agar) overlay to a final density of approximately 10^6^ PFU/ml^−1^ on top of a KB agar plate (1.2% agar) in rectangular Petri dishes (PlusPlates, Singer Instruments, UK). Phage-free lawns were also included as a control. Bacterial clones that had been incubated for 48 h at 37°C in a static humidity incubator (75% humidity) in 96-well plates were inoculated onto these agar plates using the Mast Uri^®^ dot (Mast Group Ltd, UK) with the 96-pin inoculum head and flame-sterilized 2.4 mm pins. Plates were then incubated for 16 h at 37°C in a static humidity incubator (75% relative humidity), and colonies were imaged in a PhenoBooth™ connected to associated PhenoSuite™ software (Singer instruments, UK). Colony size was quantified from plate images using MATLAB^®^ (v. R2022B, Windows). Phage resistance for each bacterial clone was measured as relative bacterial growth (RBG) by comparing colony size with versus without phage (equation (2.1) [31]); this describes how the presence of phage impacts bacterial growth, such that RBG = 1 indicates complete resistance (i.e. no change in bacterial growth in the presence versus absence of phage) and RBG = 0 indicates complete susceptibility (i.e. no bacterial growth detected).

For phage (*i*), bacteria (*j*),


(2.1) $${RBG}_{ij}=\;\frac{{\left(colony\;size\;at\;16h\right)}_{ij}}{{\left(colony\;size\;at\;16h\right)}_{controlij}}$$

BPINs were plotted using RBG bounded between 0 and 1. Binary resistance was then used to calculate summary metrics, e.g. number of phage resistances: a threshold of RBG = 0.8 was set meaning that clones with <20% reduction in bacterial growth with phages were classed as resistant.

### (d) Co-resistance network analysis

Co-resistance networks were built for each population using binary RBG values in R (v. 4.0.4) with the package igraph (v. 1.3.0) [32]: each node represents a phage; the size of nodes indicates the number of phage-resistant clones per population (out of 90), and the weight of connecting vertices indicates the number of clones with resistance to the two connected nodes. Co-resistance phage modules were determined based on edge-betweenness (R package ‘igraph’).

### (e) Quantifying efficacy of a six-phage cocktail against *Pseudomonas aeruginosa* populations

We next tested the efficacy of a six-phage cocktail to control the growth of each diverse *P. aeruginosa* population. For each population, the set of 90 *P*. *aeruginosa* clones was pooled by combining equal volumes of dense bacterial cultures, pelleted, washed and resuspended in sterile buffer (PBS, phosphate buffered saline) before storing in 20% glycerol (−80°C). Phage stocks were standardized by density then combined in equal volumes to create the six-phage cocktail (table 1). Overnight cultures of pooled bacterial populations (final density approx. 10^6^ CFU/ml^−1^) were then cultured with the phage cocktail at a range of multiplicity of infection (MOI; 100, 10, 1, 0.1 and 0.01) in 150 μl of KB media in 96-well plates; eight replicates of each combination were included, alongside positive (e.g, phage free) and negative controls (e.g. phage only). The 96-well plates were incubated without shaking at 37°C for 48 h in a plate reader that recorded optical density (OD) at 600 nm every 20 min (LogPhase 600 Microbiology Reader, BioTek, USA). Growth curves were plotted in R (v. 4.0.4) using established methods [30]. Phage cocktail efficacy (equation (2.2)) was calculated at 8 and 48 h, such that for the phage cocktail (*i*), bacteria (*j*), where (*t*) is time point (*h*),


(2.2) $${efficacy}_{t,\;ij}=\;1-\;\frac{{\left(integral\;of\;growth\;curve\right)}_{t,ij}}{{\left(integral\;of\;growth\;curve\right)}_{t,controlij}}$$

An efficacy score of 0 indicates the phage cocktail has no effect on bacterial growth (i.e. equal bacterial growth in the presence versus absence of the phage cocktail), whereas efficacy scores above 0 indicate that the phage cocktail reduced bacterial growth.

### (f) Quantifying bacterial defence system content per genome

The bacterial defence system content per genome for 160 genome sequenced clones [22] (*n* = 16 genomes per population) was previously determined using DefenseFinder [33] (data provided from Harrington *et al.* [22]. The summary metric, number of defence systems, quantifies the number of unique bacterial defence systems that were identified per isolate.

### (g) Statistical analysis

All statistical analyses were performed in R (v. 4.0.4), and the package ggplot2 [34] was used for plotting. For individual phage resistance metrics (RBG), an analysis of variance (ANOVA) with post hoc Tukey testing was used to explore the variation due to phages and between patients. The impact of defence systems on individual resistances (RBG) was investigated using a linear model, with interacting fixed effects of phage and number of defence systems. Factors influencing phage cocktail efficacy were interrogated using a stepwise linear model incorporating fixed effects of phage dose (MOI), time, number of individual phage resistances (based on binary RBG), mean strength of individual phage resistance (RBG), structure of co-resistance networks (number of modules) and number of defence systems. Higher order interactions between all fixed effects were maintained in the full linear model as this provided the best model fit based on Akaike information criterion.

## 3. Results

### (a) Bacteria–phage infection network structure varies between patients

**Figure 1.**
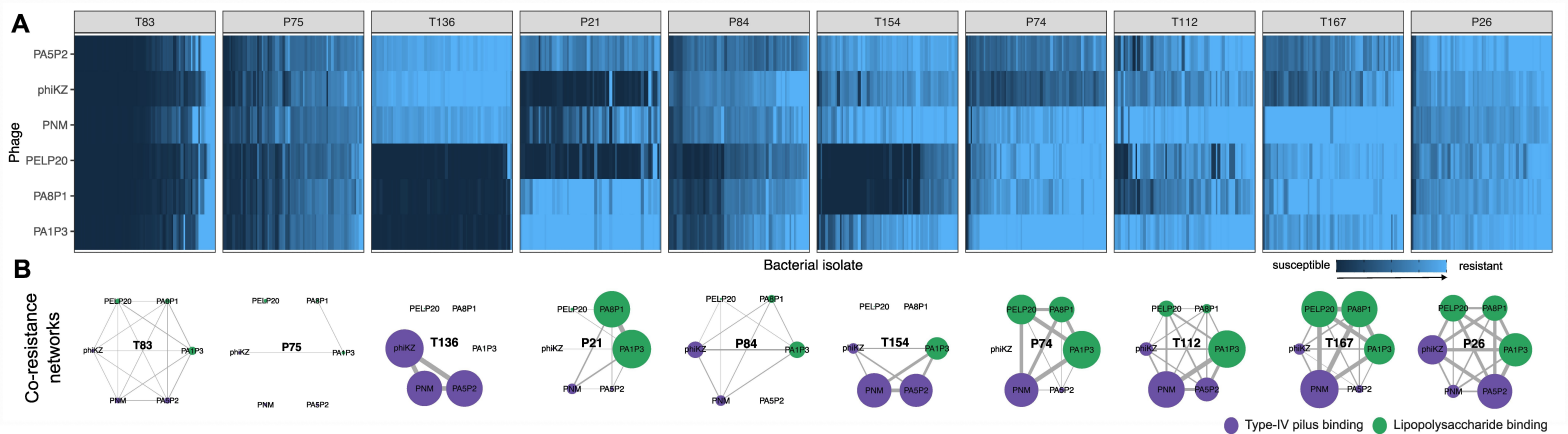
The structure of bacteria–phage infection networks and co-resistance networks. (A) Bacteria–phage infection networks describe variation in resistance between non-cystic fibrosis bronchiectasis (NCFB) patients (panel headings). Bacterial isolates (90 per patient) were challenged individually against six diverse phages: three type IV pilus binding (T4P; PA5P2, phiKZ and PNM) and three lipopolysaccharide binding (LPS; PELP20, PA8P1 and PA1P3). Resistance was quantified as relative bacterial growth in the presence versus absence of phage; see key. Networks are nested by resistance (sum of RBG) on the *x*-axis. (B) Co-resistance networks describe the frequency of binary resistance (RBG > 0.8) against each phage (node size, coloured by phage receptor: LPS in green, T4P in purple; see key) and the frequency of shared binary resistance for each phage pair (weight of connecting line). Patient network panels are ordered by total phage resistance (sum of RBG), from lowest (left) to highest (right).

To investigate the potential for phage therapy against a diverse collection of 900 *P*. *aeruginosa* clinical isolates from 10 bronchiectasis patients (90 isolates per patient) [22], we first characterized individual phage resistance profiles for each *P. aeruginosa* isolate. Resistance was quantified against a panel of six genetically and functionally diverse phages (table 1) using a novel high-throughput resistance assay. As in our previous work, phage resistance was quantified as RBG with versus without phage [31]. For each patient, we then constructed BPINs (figure 1A) to explore the diversity of phage resistance profiles both within and between patients.

Individual phage resistances varied significantly between patients (figure 1A; ANOVA_patient_ *F*_9_,_5700_ = 530.9, *p* < 0.001), ranging from very high levels of phage resistance against all phages (e.g. patient P26) to broad susceptibility (e.g. patient T83). Variation within patients was assessed by the number of unique resistance profiles. All patients contained isolates with distinct resistance profiles, but higher diversity of resistance profiles was observed in populations with higher levels of phage resistance overall (figure 1A, patients P26 and T112). This indicates that accurate profiling of phage susceptibility in chronic infections requires sampling multiple isolates per patient.

Patients with intermediate levels of overall phage resistance were more likely to have stronger phage-specific effects on isolate susceptibility (figure 1A; ANOVA_phage_ *F*_5,5691_ = 197.6, *p* < 0.001; ANOVA_phage × patient_ *F*_45,5646_ = 115.1, *p* < 0.001). To explore these phage-specific effects in more detail, co-resistance networks were built which describe the levels of shared resistances between each pair of phages (figure 1B). Patients with either very high or very low levels of overall phage resistance had uniform co-resistance networks consisting of single modules (i.e. shared resistances were as likely between all phages; figure 1A,B). However, more modularity was found within co-resistance networks for patients with intermediate levels of overall phage resistance (figure 1A,B), resulting in multiple smaller clusters of phages with higher likelihood of shared resistances. Co-resistance modules consisted of phages with shared binding receptors for some patients (e.g. figure 1B, T136 and P21). However, other modules contained phages binding different receptors (e.g. figure 1B, P74). Notably, some phages binding the same receptor also had differences in bacterial host range (e.g. PELP20 could infect a broader range of isolates than PA1P3 despite both phages targeting the LPS). Overall, this indicates that phage resistance and co-resistance were not explained by receptor binding alone, suggesting a role for additional factors such as intracellular defence systems, or other unknown bacterial resistance strategies.

### (b) Efficacy of a phage cocktail varies with bacteria–phage infection network structure and dose

**Figure 2.**
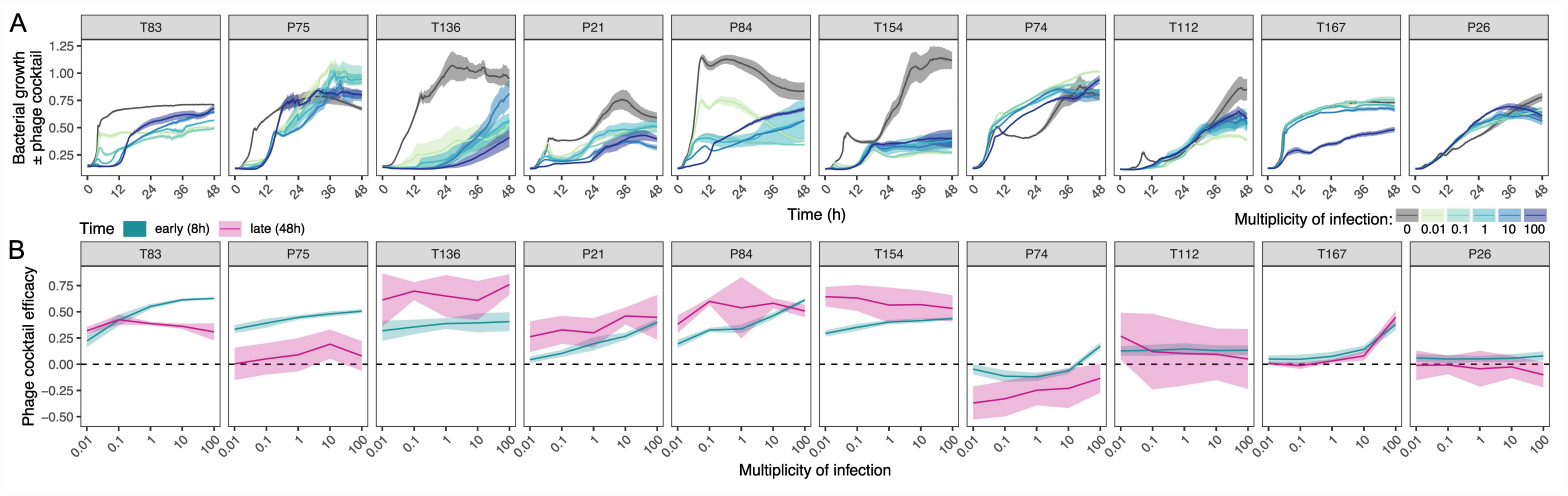
The efficacy of a six-phage cocktail against *P. aeruginosa* populations. Patient isolates (90 per patient) were pooled to create a representative bacterial population for each patient, then challenged against a six-phage cocktail containing both T4P-binding phages (PA5P2, phiKZ and PNM) and LPS-binding phages (PELP20, PA8P1 and PA1P3). (A) Bacterial growth (OD_600_) was measured every 20 min for 48 h at a range of phage doses (multiplicity of infection; see key). Plots show the mean ± s.e.m of eight replicates. (B) Efficacy of the phage cocktail (relative change in bacterial growth ± phage) was measured at an early time point (8 h) and a late time point (48 h) to observe the dose response of bacterial populations to the six-phage cocktail. Patient panels are ordered by the mean overall strength of individual phage resistances (left to right: susceptible to resistant).

Based upon these individual phage resistance profiles, we hypothesized that the efficacy of a cocktail containing all six phages would vary according to the level and structure of resistance, being most effective against those with lowest resistance (e.g. T83 and T75) but less effective as resistance increases (e.g. P26, T167 and T112). To test this, we pooled the 90 isolates per patient to form diverse bacterial populations. We tested the efficacy of the six-phage cocktail to reduce bacterial population growth over 48 h at a range of dosages (multiplicity of infection (MOI) = 0, 0.01, 0.1, 10 and 100). Phage cocktail efficacy was calculated relative to the phage-free control (figure 2A) at early (8 h) and late (48 h) time points to capture the variation in dose response over time (figure 2B). The effect of MOI was strongest at early (8 h) versus late (48 h) time points (figure 2B, LM_MOI_ *F*_1,699_ = 106.1, *p* < 0.0001; LM_MOI × time_ F_2,798_ = 27.6, *p* < 0.0001), and the dose-response also varied by patient (figure 2B, LM_MOI × patient_ *F*_10,790_ = 16.0, *p* < 0.0001). However, overall, phage cocktail efficacy varied most strongly between patients (figure 2B, LM_patient_ *F*_9,791_ = 302.9, *p* < 0.0001).

Therefore, we next explored how individual phage-resistance profiles shaped responses to the six-phage cocktail. The influence of individual resistance profiles was captured by three main effects: cocktail efficacy was limited by increasing numbers of phage resistances per isolate (linear model (LM)_number of resistances_ *F*_1,799_ = 716.53, *p* < 0.0001), increasing strength of phage resistance (LM_RBG_ *F*_1,798_ = 340.45, *p* < 0.0001) and higher co-resistance between phages (i.e. reduced modularity; LM_co-resistance modularity_ *F*_1,797_ = 108.0, *p* < 0.0001). However, over time the influence of these individual resistance metrics on cocktail efficacy reduced (LM_number of resistances × time_ *F*_2,783_ = 17.44, LM_RBG × time_ *F*_2,776_ = 53.75, LM_co-resistance modularity × time_ *F*_2,769_ = 11.75, all *p* < 0.0001).

Together, these findings indicate that both phage dose and individual phage-resistance profiles predict the immediate response to phage cocktail treatment, but these effects may weaken at later time points. Hence, simple metrics predicting efficacy can be identified that may enable phage treatment design and dosing.

### (c) Increasing phage resistance and decreasing phage cocktail efficacy with higher defence systems per genome

**Figure 3.**
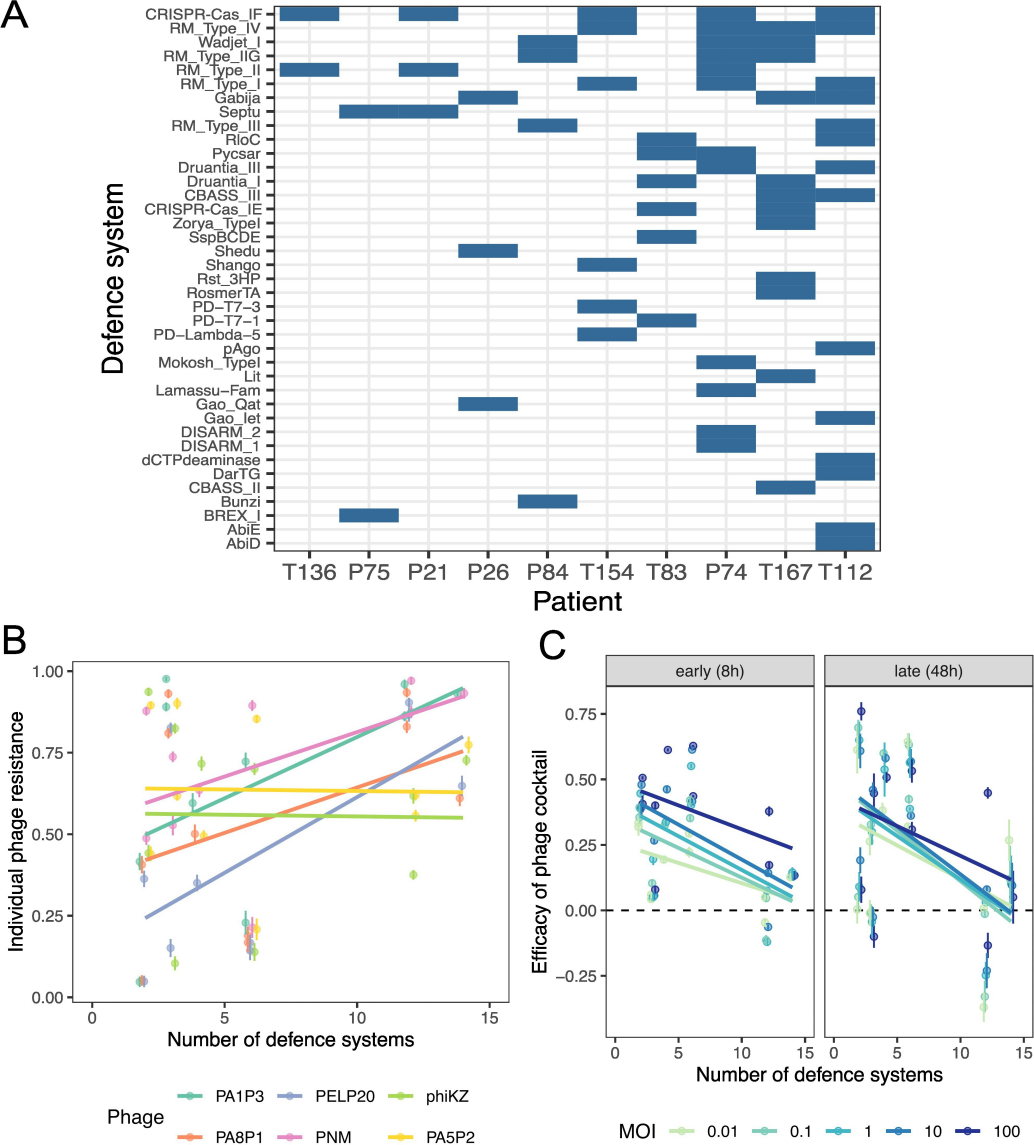
The relationship between defence system number and resistance to individual phages and the phage cocktail. (A) Bacterial defence systems were identified in all patients (16 isolates per patient, no variation within patient was observed). Total number of defence systems ranged from 2 to 14 systems per isolate and varied in identity. Patients are ordered by increasing number of defences, left to right. Defence systems are ordered by decreasing occurrence, top to bottom. (B) The impact of defence system number (as number of unique systems per isolate) on average strength of resistance to individual phages (RBG). Data points show the mean RBG (±s.e.m) for each patient’s bacterial isolates against each individual phage (see key); data are slightly jittered to limit overlap where data points are dense. (C) Efficacy of the six-phage cocktail (mean ± s.e.m) varied with defence system number (unique systems per isolate) at early (8 h) and late (48 h) time points across a range of phage doses (MOI; see key). The dashed line indicates the efficacy value at which the phage cocktail has no effect on bacterial growth (efficacy = 0).

We hypothesized that bacterial defence systems could contribute to differences in phage cocktail efficacy between bacterial strains, and thus patients. Therefore, we next identified defence system content in a subset of previously whole-genome sequenced clinical isolates (16 per patient; figure 3A) [22]. We identified multiple defence systems in all genomes. Although within-patient variation was minimal, we observed large variation between patients in both the number of systems present (ranging from 2 to 14 unique systems) and the identity of defence systems (figure 3A). CRISPR-Cas subtype I–F was the most common defence system, present in 50% of isolates. Most isolates contained at least one restriction-modification system (observed RM types included IV, IIG and I), while other common systems included Wadjet and Gabija, each identified in approximately 30% of isolates.

Overall, a higher number of defence systems increased the number of individual phage resistances (figure 3A, *F*_1,949_ = 139.9, *p* < 0.0001) and the strength of individual phage resistances (figure 3B, *F*_1,5694_ = 528.6, *p* < 0.0001). However, strength of resistance to some phages (phiKZ and PA5P2) did not increase with more defence systems (figure 3B, LM_phage × defence systems_ *F*_5,5694_ = 66.1, *p* < 0.0001). Furthermore, numbers of bacterial defence systems strongly predicted short-term efficacy of the six-phage cocktail (figure 3C, LM_defence systems_ *F*_1,794_ = 812.69, *p* < 0.0001), although this effect reduced slightly over time (figure 3C, LM_defence systems × time_ *F*_1,783_ = 26.4, *p* < 0.0001), it was maintained across phage doses (figure 3C). This indicates that genetic sequencing of patient infections to identify bacterial defence system content is a valuable addition to individual resistance phenotyping to predict appropriate phage cocktails.

## 4. Discussion

In chronic *P. aeruginosa* respiratory infections, the high strain diversity between patients and rapid phenotypic and genetic diversification of populations within infections are likely to pose serious challenges for developing phage therapeutics [21–24]. Here, we show that simple BPIN metrics can explain the efficacy of a multi-phage cocktail against diverse *P. aeruginosa* populations isolated from bronchiectasis lung infections. Specifically, increasing per isolate resistance, stronger resistance and lower modularity of co-resistance all weakened cocktail efficacy. Further, we show that a higher number of anti-phage defence systems per genome increased the strength of resistance to some of the phages and, importantly, decreased the efficacy of the phage cocktail. Together, our findings highlight the importance of considering both between and within patient diversity in phage resistance and identify key BPIN metrics and genomic features that could be used to guide design of phage therapeutics against *P. aeruginosa* chronic infections.

Because *P. aeruginosa* is an opportunistic pathogen, strains tend to vary between patients in CF and bronchiectasis chronic infections, with epidemic clones being uncommon [21,22]. Each of the patient infections studied here was caused by a single, distinct sequence type [22]. Such high between-patient strain variability drove strong differences in BPIN structure and properties among patients, which in turn caused varying efficacy of the phage cocktail. Unlike pathogens with a strong epidemic population structure, it seems unlikely, therefore, that standard ‘off the peg’ phage cocktails will be appropriate treatments for *P. aeruginosa* chronic infections, requiring instead a more personalized approach. Given the long duration of infection, the relative stability of strains within such infections, as well as the intensive clinical monitoring of these patients, personalized phage therapy may be a realistic prospect [35–37]. Herein, the infecting strain can be characterized and phage susceptibility tested as part of routine clinical monitoring, potentially allowing time for phage cocktails to be designed for later use during exacerbations.

Chronic lung infections typically undergo extensive phenotypic and genetic diversification as populations adapt to the lung environment [23,24]. Here, we show that this diversification in turn causes extensive within-population diversity in phage-resistance profiles among isolates from the same patient belonging to the same strain. All populations included multiple distinct resistance profiles, with the number of distinct resistance profiles increasing with the overall level of resistance. Traits commonly undergoing diversification within chronic lung infections are often those acting as targets for phage adsorption, including motility structures, such as type-IV pilus or flagellum, and components of the cell envelope, such as lipopolysaccharide or membrane proteins [22,25]. Such diversification is likely driven by a wide variety of selective forces, including lung microenvironments, antibiotic treatments, host immunity or the presence of hypermutator strains [21,22,38,39] but may also be caused by phages naturally present in the lung. Free virions have been detected at high abundance in lung sputum from cystic fibrosis patients, most likely through induction of lysis by temperate phages integrated into bacterial genome(s) [40]. Moreover, free temperate phages have been shown to select for *P. aeruginosa* resistance mutations in type-IV pilus genes in sputum-mimicking media [41]. Hypermutator strains could further accelerate diversification and resistance evolution, and their role in shaping phage resistance dynamics represents an important avenue for future study.

The structure of within population diversity in phage resistance can be described using BPINs [42]. BPINs have been widely used to understand the ecology and evolution of natural bacteria–phage communities [43–46]. However, BPINs have less often been applied to understand phage cocktail efficacy. Consistent with a recent computational study, our data suggest that simple BPIN metrics are likely to be valuable for guiding phage cocktail design [47]. Unsurprisingly, we found that minimizing the number and strength of resistances maximizes the efficacy of the phage cocktail. Importantly, however, we also show that phage cocktails can work well even when there are relatively high numbers of strong resistances, provided that the co-resistance structure is modular. In practice, this may mean that complex phage cocktails can be readily simplified without losing efficacy by removing the phages forming co-resistance modules, potentially simplifying the manufacture and regulatory approval process. As such, co-resistance networks may be a useful addition to the design toolkit for phage therapeutics, in addition to standard BPINs. Interestingly, co-resistance modules were not solely structured by shared receptor use, suggesting that other aspects of phage life history and bacterial defence play a role in shaping infection outcome.

The genomic content of anti-phage defence systems was positively correlated with the strength of resistance to some of the individual phages. Notably, infection by two of the phages, phiKZ and PA5P2, was unaffected by the number of defence systems per genome. In the case of the jumbo phage phiKZ, this is likely to be due to its production of a nuclear shell, protecting its genome against many bacterial defence systems, including restriction-modification and CRISPR-Cas [48], which were among the most common defence systems here. An increasing breadth of individual phage resistances with increasing number of defence systems per genome is consistent with previous findings [27]. Despite this overall trend, there were notable outliers: for example, the strain infecting patient P26 had only three defence systems, but the most individual phage resistances, highlighting that infection requires both successful adsorption and evasion of intracellular defence systems. In contrast, the strain infecting patient T83 had six defence systems, but the weakest level of phage resistance, suggesting that defence system identity as well as number are important for infection outcome. Importantly, in addition to individual resistances, we show here that a higher number of defences per genome reduced the efficacy of a multi-phage cocktail to control the population. This relationship suggests that strains with lower numbers of defence systems are likely to be particularly good candidates for phage therapy. Our approach combines individual-level phenotypic resistance testing with genomic profiling, highlighting how both *in vitro* and *in silico* data can be integrated for more effective phage cocktail design.

The relationship between dose and efficacy varied between patients and was stronger at the 8 h sampling point, such that for some patients, higher dose led to greater initial control of bacterial population growth. Interestingly, a lack of positive relationship between dose and efficacy was not only seen for cases where the phage cocktail was ineffective (e.g. P74, T112 and P26) but also for cases where bacterial growth was suppressed equivalently well irrespective of dose (e.g. T154 and T136). These latter cases potentially suggest that *in situ* replication of phages (i.e. phage auto-dosing) may enable low-dose phage cocktails to attain high efficacy even against diversified bacterial populations. In one patient (T167), we observed that the phage cocktail became effective only at very high dose (i.e. MOI = 100), despite high per isolate resistance to individual phages and low co-resistance modularity, suggesting that high-level resistance can be overcome by high dosing in some cases. Together, these findings suggest that higher phage doses are likely to be more generally effective but not universally so.

In conclusion, we show that diversified *P. aeruginosa* populations typical of chronic infections are amenable to treatment with phage therapy. However, because infecting strains vary between patients, standard ‘off the peg’ phage cocktails are unlikely to offer broadscale effectiveness, requiring more personalised approaches to phage therapeutic design. Given the chronic nature of many *P. aeruginosa* infections, the relative stability of infecting strains within such individuals and intensive clinical monitoring, sequencing of clinical isolates as part of routine care is becoming increasingly feasible [14,16]. Falling costs and shorter turnaround times for pathogen genome sequencing are likely to drive even more widespread clinical adoption in future, allowing for this information to be used in the design of tailored phage therapies for individual patients [49]. Broad implementation would require automated sequencing and data analysis workflows to make the process cost-effective and time-efficient in the clinical setting. The simple BPIN and co-resistance network metrics could help to guide personalized phage cocktail design, as well as a simple rule of thumb that strains with lower numbers of defence systems per genome tend to be good candidates for phage therapy.

## Data Availability

All newly collected data associated with the paper have been uploaded as electronic supplementary files. Supplementary material is available online [50].
